# Supplementary material for: Nutritional Content and Microbial Load of Fresh Liang, Gnetum gnemon var. tenerum Leaves
Source: Foods. 2023 Oct 20;12(20):3848. doi: 10.3390/foods12203848 (PMC10605991; doi:10.3390/foods12203848)
Supplement: Supplementary file 1 [file foods-12-03848-s001.zip › foods-2615700-supplementary.pdf]

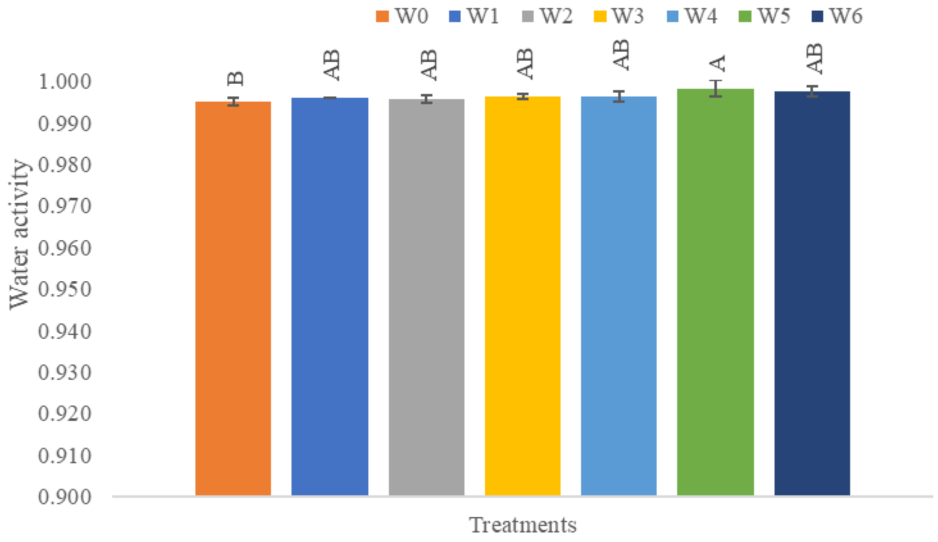

Figure S1. water activity of liang leaves added with distilled water at 0-41.18%. W0 means liang leaves without water added; W1 means liang leaves added with water at 16.67%; W2 means liang leaves added with water at 23.08%; W3 means liang leaves added with water at 28.57%; W4 means liang leaves added with water at 33.33%; W5 means liang leaves added with water at 37.5%; W6 means liang leaves added with water at 41.18%.
